# Health professionals’ knowledge and attitudes towards baby led weaning: A cross-sectional, exploratory, and observational study

**DOI:** 10.1371/journal.pone.0334768

**Published:** 2026-03-18

**Authors:** Paula Sarreira-de-Oliveira, Renata Ramalho, Ricardo Antunes, Vanessa Antunes, Fernanda Loureiro

**Affiliations:** 1 Egas Moniz Center for Interdisciplinary Research (CiiEM), Egas Moniz School of Health & Science, Almada, Portugal; 2 Egas Moniz School of Health and Science, Nutritional Immunology – clinical and experimental Lab (NICE Lab), Clinical Research Unit, Egas Moniz Center for Interdisciplinary Research (CiiEM), Almada, Portugal; 3 Universidade Católica Portuguesa, Centro de Investigação Interdisciplinar em Saúde (CIIS), Lisboa, Portugal; Kermanshah University of Medical Sciences, IRAN, ISLAMIC REPUBLIC OF

## Abstract

Baby Led Weaning (BLW) is a type of infant feeding that allows babies to feed themselves and has gain popularity among parents in recent years. Health professionals’ (HP) knowledge and attitudes towards BLW is less explored. We conducted a cross-sectional, exploratory, and observational study, with a web-based instrument, that aimed to determine the knowledge and attitudes of HP familiar to BLW regarding the use of this method in complementary feeding. In our sample of 118 HP, HP who were women or younger (23–39 years) had a more positive perception of BLW. We found that acceptance and positive attitudes towards BLW were consistent among HP. It is essential to improve the training of HP in the specifics of implementing this approach and to develop clear guidelines to guide practice.

## Introduction

From the age of six months, international guidelines recommend the introduction of complementary foods of different textures, flavours and appearances alongside breast milk [1]. Breastfeeding, which should continue until 2 years of age or beyond [2], or formula feeding, as the baby is introduced to complementary foods, is gradually reduced during this period. The introduction of complementary feeding is a crucial stage of growth for children that has long-term effects on physical, cognitive, and socio-emotional well-being [3]. Given the potential influence that the methods for introducing complementary feeding can have on developmental domains, this transition period has drawn substantial attention. One such approach is Baby Led Weaning (BLW) which is recognized for promoting independence, helping to regulate food intake, and can encourage healthier eating habits in a safe environment [4]. BLW is gaining popularity in scientific literature [5]. Supporters argue that it fosters autonomy, develops motor skills, and positively impacts eating habits and acceptance of healthy foods [6]. Recent studies have investigated the BLW method, namely its effect on the risk of choking [7,8], iron status [9], anemia and obesity [7] and concluded that the BLW does not pose any risks compared to the traditional approach. However, most authors state the dearth of sufficient evidence, which has led to the lack of internationally accepted guidelines or their recommendation of the BLW method over traditional forms of introducing complementary feeding [10].

Literature on BLW has focused on how it’s implemented, how it affects children’s health, the perspectives of those involved (parents and health professionals [HP]) and/or a mixture of the above. Of these areas, the least studied are the perspectives of HP [11,12], even though their influence on parents’ choice of methods for introducing complementary feeding is recognized [13].

HP, including nurses, dietitians, pediatricians, and general practitioners, play a crucial role in promoting safe feeding practices. Improved education and increased research into BLW are essential to provide evidence-based guidance, helping parents make informed decisions about their children’s nutrition and development. BLW remains as scientifically under-researched subject [12]. Authors agree that there is a lack of sufficient studies, both on the method itself [5,10] and, more specifically, on HP perspective [11,12]. We aim to determine the knowledge and attitudes of HP familiar to BLW regarding the use of this method in complementary feeding. Additionally, we seek to evaluate how socio-demographic characteristics influence HP’ knowledge and attitudes towards BLW.

## Materials and methods

### Study design

This was a cross-sectional, exploratory, and observational study [14] that adhered to the reporting guidelines of the Enhancing the Quality and Transparency of Health Research (EQUATOR) network. The study followed specifically the Strengthening the Reporting of Observational Studies in Epidemiology (STROBE) checklist for cross-sectional studies and was registered at Open Science Framework (https://osf.io/8bcw3/).

### Setting

The study population consisted of HP from the Regional Health Administration of Lisboa and Tagus Valley, covering 15 health units across a large urban area in Portugal. According to official regional data, a total of 5,613 healthcare professionals are employed in this region.

### Eligibility criteria

For the purposes of this study, only those working in primary healthcare and directly involved in child nutrition were considered eligible. However, the exact number of professionals who meet these specific criteria is not available, which limits the ability to determine a precise response rate. To ensure participants met the inclusion criteria, the first question asked was: “Are you directly or indirectly involved in child nutrition (e.g., through conducting consultations for child health surveillance)?”. Individuals who were not involved in child nutrition and unfamiliar with the BLW method were excluded from the study.

### Variables

In this study, we collected sociodemographic data (sex, age, professional category, qualifications, and years of professional experience), as well as variables related to practices, knowledge, and attitudes toward BLW, which are further explained in the following section.

### Data sources

We began by conducting a literature review [12], which highlighted a lack of evidence on this topic and helped us identify a previously used instrument for assessing HP knowledge and attitudes [15–17]. Since it was not available in the language were the study aimed to be implemented, we performed the translation, cross-cultural validation and determined the psychometric properties of HP’ knowledge and attitudes towards the BLW instrument into European Portuguese [18]*.*

This is a self-administered instrument that was made available online for participants’ convenience and ease of access. It includes a total of 25 closed-ended questions regarding the approach to introducing complementary feeding and assessing familiarity with the BLW method. The instrument comprises a section designed to collect socio-demographic data, a section regarding practices and a section with 12 questions, rated on a 5-point scale, that are specifically targeted at the knowledge and attitudes of HP. This section is structured into three dimensions: (1) child-centered contributions, addressing the advantages of the BLW approach for the child; (2) parent-centered contributions, reflecting perceived benefits for parents; and (3) constraints, referring to potential challenges or concerns associated with BLW (such as worries about insufficient weight gain). All dimensions demonstrated good internal consistency, with Cronbach’s alpha values exceeding 0.70. The translation and validation process demonstrated that the instrument is a reliable tool for assessing healthcare professionals’ knowledge and attitudes toward BLW [18].

### Bias

To minimize selection bias, the study included all HP working in primary healthcare who were potentially involved in child nutrition, regardless of their specific professional category or years of experience. Recruitment was conducted through institutional channels to ensure that all eligible HP had the opportunity to participate, rather than targeting specific individuals or groups. Participation was voluntary, and no incentives were offered, reducing the likelihood of coercion or selective recruitment. Additionally, anonymity of responses was guaranteed, which aimed to reduce social desirability bias and encourage uninfluenced reporting of knowledge, practices, and attitudes.

Regarding information bias, the questionnaire was based on an instrument previously used which we translated and validated. This approach helped ensure that items were clearly formulated and consistently interpreted by participants. Data collection procedures were standardized, and all responses were self-reported using a digital platform, minimizing interviewer influence.

### Sample size

Sample was of convenience type. An email was sent to all HP via professional electronic mail inviting them to participate in the study. The invitation included information about the aim and scope of the study, the requirements for participation and a direct link to the instrument. This approach ensured that all potential participants were contacted and were able to participate. The instrument was applied over a three-month period, from 1 October to 31 December 2023.

### Quantitative variables

Data were organized and classified using statistical treatment based on the IBM SPSS Statistics® for windows, v. 27.0. IBM Corp. Released 2020, Armonk, NY, USA. All variables were analyzed using descriptive statistics. Categorical variables were presented as numbers and percentages, and continuous variables as mean and standard deviation.

### Statistical methods

As appropriate, statistical significance was tested for associations between variables using ANOVA test. In addition, the normality of the data was verified using the Kolmogorov-Smirnov test, and the homogeneity of variances using the Levene test. P-values < 0.05 were considered statistically significant. Inferential analysis was performed with independent variables (sex; age; profession; being aware of BLW benefits; recommending BLW; and witnessing a child eating according to the BLW method) and dependent variables (knowledge and attitudes of HP towards the BLW method).

### Ethical issues

The study received approval from the Technical and Scientific Council on February 1, 2023, and from the Ethics Committee on March 30, 2023 (approval number 1213.23). Given that the study was conducted in a particular institution, it was also subjected to the review and approval of the Ethics Committee of this institution. The approval was given on September 15, 2023, with approval number 50297/2023. The online questionnaire comprised a description of the study, the purpose of the participation, and a solicitation for informed consent. The document indicated that participation in the study was optional, and that the healthcare professional had the right to resign from the study at any given point. Furthermore, it guaranteed the preservation of anonymity, confidentiality, and the protection of data. HP could only enroll if they were willing to participate in the study and gave their written consent.

## Results

**Fig 1:**
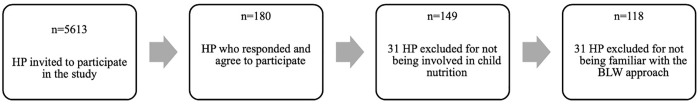
Flow diagram on sample recruitment and exclusion.

Initially, 180 HPs participated in our study, but the application of inclusion/exclusion criteria resulted in a final sample size of n = 118, as described in Fig 1.

Table 1 details the sample distribution regarding socio-demographic characteristics and general knowledge about the BLW method.

**Table 1:** Participants’ socio-demographic characteristics and general knowledge about BLW.

Variables	n	%
**Sex**		
Male	16	13.6
Female	102	86.4
**Age Groups**		
23-39 years	28	23.7
40-49 years	43	36.4
>=50 years	47	39.8
**What is your professional category?**		
Nurse	92	78.0
Nutritionist/ Dietitian	15	12.7
Other health professional	11	9.3
What was your primary source of information on BLW?		
The parents/ caregivers themselves	22	18.6
Training (course, lecture/seminar/conference, etc.)	35	29.7
Website	29	24.6
Other healthcare professionals	28	23.7
Scientific literature	2	1.7
Other	2	1.7
**Aware of the benefits of the BLW method?**		
Yes	102	86.4
No	16	13.6
**Recommend the practice of the BLW method?**		
Never	28	23.7
Sometimes	58	49.2
Frequently	29	24.6
Always	3	2.5
**Have you witnessed children eating food according to the BLW method?**		
Yes	63	53.4
No	55	46.6

Most respondents were female (86.4%) and nurses (78%). The age distribution reveals a relatively older population, with 39.8% being 50 years or older. The sources of information on this method were relatively evenly distributed among training, websites, other healthcare professionals, and the parents or caregivers themselves.

Most respondents were aware of the benefits of the BLW method (86.4%). Only a small portion of respondents (23.7%) had never recommended the application of this method, and 53.4% had already witnessed children consuming food according to the BLW method. The results of the inferential analysis with independent and dependent variables are presented in Table 2.

**Table 2:** Variables statistical correlation analysis between socio-demographic and general knowledge about BLW and the dimensions of the instrument.

	Positive child-centered contributions (range 1–5)			Positive parent-centered contributions (range 1–5)			BLW constraints (range 1–5)		
Variables	Mean	SD	p	Mean	SD	p	Mean	SD	p
**Sex**									
Male	3.5	0.53	<0.001	3.2	0.42	0.025	3.1	0.66	0.304
Female	4.3	0.59		3.7	0.74		2.8	1.17	
**Age Groups**									
23-39	4.6	0.46	<0.001	3.9	0.62	0.023	2.5	1.28	0.029
40-49	4.0	0.71		3.4	0.76		2.7	0.98	
>=50	4.1	0.56		3.6	0.70		3.1	1.07	
**Profession**									
Nurse	4.2	0.61	0.537^a^	3.6	0.69	0.583^a^	2.9	1.07	0.064^a^
Nutritionist	4.4	0.74		3.7	0.78		2.2	1.13	
Other Health Prof.	4.1	0.72		3.8	0.99		3.0	1.34	
**Aware of the benefits of BLW?**									
Yes	4.3	0.58	<0.001	3.7	0.73	0.025	2.8	1.14	0.432
No	3.5	0.53		3.2	0.55		3.0	0.96	
**Do you usually recommend BLW?**									
Never	3.6	0.68	<0.001	3.1	0.73	<0.001	3.1	0.77	0.002
Sometimes	4.3	0.47		3.6	0.66		3.0	1.12	
Frequently	4.5	0.53		4.0	0.58		2.3	1.20	
Always	4.7	0.17		3.8	0.52		1.3	0.58	
**Have you witnessed a child eating according to the BLW method?**									
Yes	4.3	0.50	0.004	3.7	0.66	0.046	2.9	1.13	0.628
No	4.0	0.72		3.5	0.77		2.8	1.10	

^a^Kruskal-Wallis test.

Among the three constituent dimensions, *positive child-centred contributions* showed the highest mean values, followed by *positive parent-centred contributions*. In contrast, mean scores for *BLW constraints* were lower, indicating fewer perceived difficulties associated with the method.

The variable profession did not present a normal distribution; therefore, the non-parametric Kruskal-Wallis test was applied. No statistically significant differences were observed among professional groups across the three dimensions. For all other variables, assumptions of normality and homogeneity of variance were met, and one-way ANOVA tests were performed. Partial eta squared (η²ₚ) and observed power (π) were also computed.

The sex variable proved to be statistically significant in relation to the *positive child-centred contributions* dimension F (1,118) = 22.792; p < 0.001; η²ₚ = 0.164; π = 0.997) with female HP demonstrating a more positive attitude (M = 4.3; SD = 0.59) compared with male HP (M = 3.5; SD = 0.53). In the *positive parent-centred contributions* dimension (F (2, 118) = 5.187; p < 0.025; η²p = 0.043; π = 0.617), women also demonstrated more positive attitudes (M = 3.7; SD = 0.74) than male HP (M = 3.2; SD = 0.42).

Age emerged as a differentiating factor across all three dimensions, in *positive child-centred contributions* (F (2,118) = 7.517; p < 0.001; η²ₚ = 0.116; π = 0.939), in *positive parent-centred contributions* (F (2,118) = 3.886; p = 0.023; η²ₚ = 0.063; π = 0.692) and in *BLW constraints* (F (2,118) = 3.662; p = 0.029; η²ₚ = 0.060; π = 0.664). A post hoc Tukey HSD test revealed significant that younger participants (23–39 years) reported higher *positive child-centred contributions* than those aged 40–49 years (p < 0.001) and ≥ 50 years (p = 0.011). Similarly, they reported higher *positive parent-centred contributions* than the 40–49 group (p = 0.017). Regarding *BLW constraints*, younger participants perceived fewer constraints than those aged ≥ 50 years (p = 0.029). No other pairwise comparisons reached statistical significance.

Participants who were aware of the benefits of BLW exhibited a significantly more favourable representation of its implementation (F (1,118) = 25.560; p < 0.001, η²ₚ = 0.181; π = 0.999), reporting higher *positive child-centred contributions* (M = 4.3; SD = 0.58) and higher *positive parent-centred contributions* (F (1,118) = 5.187; p = 0.025; η²ₚ = 0.043; π = 0.617; M = 3.7; SD = 0.73). Similarly, health professionals who frequently or always recommend BLW demonstrated a more positive perception and less apprehension regarding its implementation in children (F (3,118) = 15.433; p < 0.001; η²ₚ = 0.289; π = 0.999), reported greater *positive parent-centred contributions* (F (3,118) = 9.466; p < 0.001; η²ₚ = 0.199; π = 0.997), and perceived fewer *BLW constraints* (F (3,118) = 5.389; p = 0.002; η²ₚ = 0.124; π = 0.928).

A Tukey HSD test was performed to examine differences in the three dependent variables. For *positive child-centred contributions*, significant differences were found between those who *never* recommend BLW and all other groups – *sometimes* (p < 0.001), *frequently* (p < 0.001), and *always* (p = 0.009) – indicating that those who never recommend BLW reported markedly lower scores. For *positive parent-centred contributions*, participants who *never* recommend BLW scored significantly lower than those who *sometimes* (p = 0.008) or *frequently* (p < 0.001) recommend it. Additionally, those who *sometimes* recommend BLW scored higher than those who *frequently* do (p = 0.025).

Regarding *BLW constraints*, significant differences were observed between those who *never* recommend BLW and both the *frequently* (p = 0.044) and *always* (p = 0.039) groups, as well as between the *sometimes* and *frequently* (p = 0.021) and *always* (p = 0.038) groups. Overall, participants who more often recommend BLW perceived fewer constraints related to the method.

HP who had observed a child eating according to the BLW method evaluated it as more beneficial in terms of *positive child-centred contributions* (F (1, 118) = 8.510; p = .004; η²p = 0.068; π = 0.825; M = 4.3; SD = 0.50) and *positive parent-centred contributions* (F (1, 118) = 4.084; p = 0.046; η²p = 0.034; π = 0.518; M = 3.7; SD = 0.66). Finally, HP who had witnessed a child eating according to the BLW method evaluated it as more beneficial in a *positive child-centred contributions* (F (1,118) = 8.510; p = 0.004; η²ₚ = 0.068; π = 0.825; M = 4.3; SD = 0.50) and in greater *positive parent-centred contributions* (F (1,118) = 4.084; p = 0.046; η²ₚ = 0.034; π = 0.518; M = 3.7; SD = 0.66).

## Discussion

This study examined the knowledge and practices of HP already familiar with the BLW method. Findings suggest that these professionals generally hold a positive attitude toward BLW and recommend it as a strategy for introducing complementary feeding. This favorable perspective may be influenced by the perceived benefits of the method, such as promoting motor development, encouraging infant autonomy, and greater likelihood of developing a diverse and balanced diet [12]. These advantages are believed to contribute to key developmental milestones and foster healthy eating habits in early childhood. The literature also suggests that in addition to these benefits, BLW is consistent with the introduction of solid foods when the baby shows signs of readiness, promotes food variety, and help with early exposure to different textures, which supports the development of chewing skills and acceptance of new foods [19]. Also, BLW promotes respect for hunger and satiety cues, which helps prevent overfeeding and supports intuitive eating [15,19–21].

Among HP who accepted to participate in our study and were involved in child nutrition, 31 were not aware of the BLW method as Fig 1 details. This highlights a gap in professional training and practice that does not include this approach to infant feeding. Continuing education is crucial to ensure professionals are aware of new practices and research in child nutrition [11]. This lack of knowledge not only affects the quality of guidance offered to families but can also impact children’s health and development. Uninformed professionals may discourage parents from exploring this approach, even though it has benefits for both parents and children. As the children play an active role in the use of this approach, it has advantages over the traditional method in that it gives them greater involvement in their own feeding. The absence of accurate information can create confusion among parents, who may find contradictory information in unreliable sources such as social networks and blogs [22,23]. This misinformation can lead to unsafe eating practices and, consequently, long-term health problems [24].

Our results highlight that HP who are women have a more positive attitude than HP who are men towards the BLW method, which may be attributed to social, cultural and psychological factors that shape the childcare experience. Historically, women have been associated with childcare and nutrition [25], which may result in a greater predisposition to adopt methods that emphasize children’s autonomy and health even among HP. Furthermore, literature has established that caregivers who are women, especially mothers, often seek information about child nutrition and childcare in support groups [26], social networks [27] and specialized literature [28]. It is reasonable to hypothesize that a similar pattern occurs among female HPs, where a more active search for information may contribute to a more positive attitude towards the BLW method, as our results suggest.

Several recent studies [11,15] and a 2024 scoping review [12] show that HP’ awareness of BLW is high but recommendations vary, often reflecting differences in perceived safety, training, and information sources. In mixed-methods and qualitative studies, parents commonly report learning about BLW through informal and social-media channels rather than only through HP advice, and HPs identify limited training and concerns about safety as reasons for cautious recommendations [11,12,15].

In our study, the sample included health professionals of different ages and genders; however, we observed that female and younger HPs (23–39 years) tended to demonstrate a more positive attitude toward the BLW method compared with their older or male counterparts. Although few studies explicitly test HP age or gender as primary predictors of attitudes toward BLW, literature on generational differences and technology use indicates that younger clinicians and recent graduates are more engaged with digital media and are often more open to adopting novel practices [29,30]. Together, these findings support the interpretation that sociocultural factors, broader access to online information, and evolving family dynamics may contribute to younger and female HPs holding more positive attitudes toward BLW compared with older colleagues [1,15,31].

In our sample, acceptance of and positive attitudes toward BLW were similar across healthcare professionals, regardless of their specific role: nurses; nutritionists; and other HP. This may be attributed to growing evidence supporting the benefits of this approach. Scientific consensus can facilitate communication between professionals, allowing them to offer cohesive and well-founded guidance to families, regardless of their area of intervention. When different professionals share a common vision, families receive consistent and reinforced information about food introduction. This not only improves parents’ understanding of the method but also contributes to the implementation of safe and effective feeding practices. Furthermore, it can encourage interprofessional collaboration, promoting more holistic and family-centered care. However, studies indicate that there is still a lack of comprehensive understanding and conflicting information about BLW, and that there is still a great need for research in this area [32]. Some authors even mention that, for this reason, there is still a limited percentage of professionals who recommend the method [16,17]. Our results differ from these findings, probably because only HP familiar with the BLW approach were included. Including all HP, knowledge or not regarding BLW, could lead to different results.

The growing popularity of BLW represents a significant shift from traditional spoon-feeding practices toward a more infant-led, autonomy-based approach. From a public health perspective, this shift underscores the importance of ensuring that families receive accurate, evidence-based information to minimize potential risks, while optimizing benefits related to self-regulation and dietary diversity [10,14,16]. Consequently, the increasing adoption of BLW demands that healthcare professionals adapt their counseling to evolving parental preferences and emerging evidence. The literature highlights the need for clear, standardized guidance and further research on the safe and effective implementation of this method [10]. Continuing professional education and additional investigation into the long-term outcomes of BLW are therefore essential to ensure that healthcare professionals can provide consistent, evidence-informed support to families [14]. A balanced and informed approach that acknowledges both the benefits and potential risks of BLW will contribute to promoting healthy, safe, and developmentally appropriate feeding practices from the earliest stages of life.

### Limitations

In our study, we specifically included HP with prior knowledge of BLW, enabling us to focus directly on their understanding and attitudes, which aligns with the primary objective of our research. This exclusion criterion may have influenced our findings by reducing the overall sample size, limiting the diversity of professional perspectives, and potentially over-representing individuals with prior knowledge of BLW. Consequently, the findings may reflect a more informed or engaged subset of HP. The study was planned to be disseminated throughout a major health care institution, and we anticipated a larger sample size. However, the implementation of this research coincided with a period of significant organizational change. As a result, there were changes in the e-mails that had an impact on the disclosure of the study and, consequently, on our sample size. Moreover, the use of a convenience sampling approach restricts the extent to which the findings can be generalized. To strengthen external validity, future studies should consider employing probability-based sampling techniques or conducting multicenter research across different regions or countries. The instrument used is still in its early stages of implementation and, while it provides valuable insights, it has room for improvement. Enhancements to its design and structure could lead to a more comprehensive and accurate assessment of HP knowledge and attitudes. Refining the instrument, perhaps by expanding its scope or adjusting its questions, would allow for a more nuanced understanding of HPs’ perspectives. This process of continual development is crucial to ensure that the instrument effectively captures the complexities of HP attitudes and knowledge in this area.

## Conclusions

Our findings suggest that regarding HP, women and younger age groups tend to have a more positive perception of the BLW method. No significant differences were observed between different types of HP, suggesting that BLW is similarly accepted across professions. The growing popularity of BLW reflects an ongoing shift in infant feeding practices toward more autonomy-based approaches, underscoring the need for healthcare systems to adapt to these changes. Clear, evidence-based guidelines are essential to ensure consistent and safe recommendations for families. Furthermore, sustained professional training and continuous access to updated evidence will enable health professionals to provide confident, informed, and standardized support for families adopting BLW.

## Supporting information

S1 File Health professionals’ knowledge and attitudes towards the Baby-Led Weaning instrument. (DOCX)

S2 File STROBE cross-sectional guidelines: reporting checklist. (DOCX)
